# Ayurvedic Medicinal Plants and Plant-Derived Extracellular Vesicles: Current Evidence and Future Perspectives

**DOI:** 10.3390/nano16080483

**Published:** 2026-04-18

**Authors:** Manasi Bhabal, Tiziana Pietrangelo, Mariantonia Logozzi, Stefano Fais

**Affiliations:** 1 Exolab Italia S.R.L., Technopolo d’Abruzzo, 67100 L’Aquila, Italy; manasi@exolabitalia.com; 2 Department of Neuroscience, Imaging and Clinical Sciences, Università degli Studi “Gabriele d’Annunzio”, 66100 Chieti, Italy; tiziana.pietrangelo@unich.it

**Keywords:** plant derived extracellular vesicles (PDEVs), Ayurvedic plants, therapeutics, phyto active

## Abstract

Plant-derived extracellular vesicles (PDEVs) are nanoscale carriers produced through conserved plant mechanisms, including multivesicular body (MVB) formation and consequent extracellular vesicle release. MVBs are formed through repeated rounds of intracellular vesicles’ fusion, thus leading to the incorporation into PDEVs of lipids, proteins, miRNAs, nucleic acids, and secondary metabolites, derived from different cellular compartments. PDEVs possess a bilayer lipid membrane, which protects their cargo from degradation and facilitates membrane–membrane fusion with target cells. Ayurvedic medicinal plants are renowned for their extensive phytochemical diversity and enduring efficacy in addressing inflammation, infections, metabolic disorders, cancer, and neurodegeneration. However, the clinical translation of traditional herbal preparation is severely bottlenecked by batch-to-batch variability, restricted compound bioavailability, mechanistic uncertainties, and limitations of conventional large-scale extractions. This perspective research study critically proposes PDEVs as an innovative interpretation for Ayurvedic medicinal plants utilization. We identify and evaluate medicinal plants with established therapeutic characteristics that remain unexamined in PDEV research, hence presenting compelling opportunities for future investigation. By establishing a synergistic bridge between ancient Ayurvedic knowledge and modern nanomedicine, this perspective provides a methodological roadmap to guide health-efficient plant selection and accelerate translational research in next-generation therapeutics.

## 1. Introduction

**Figure 1:**
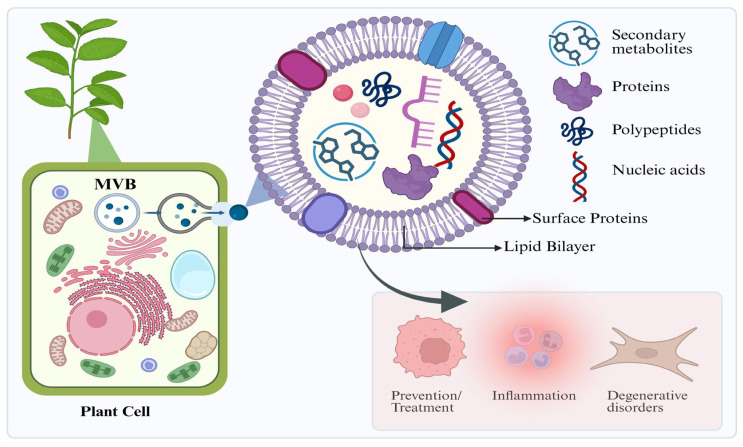
Biogenesis and structural composition of PDEVs.

Extracellular vesicles (EVs) are a family of vesicles released extracellularly with a size ranging from nano to micrometers in average diameter. They are characterized by a lipid bilayer structure, and, to date, they lack the ability to self-replicate. They can also be classified through their biogenesis and physical traits [1]. Exosomes are nanovesicles derived from repeated rounds of fusion between endosomal vesicles; then, they are released upon the fusion of multivesicular bodies with the plasma membrane. Proteins, lipids, nucleic acids, and metabolites are among the bioactive substances that are normally carried by these vesicles [2]. Figure 1 shows a representation of the biogenesis and composition of PDEVs. These molecules not only reflect the properties of the parent cell but also act as an intercellular communicator to alter physiological and pathological processes in the recipient cells [3,4,5,6]. The cells of all living beings release EVs, including plant cells.

In the last decade, a great deal of interest in plant-derived extracellular vesicles (PDEVs) has been observed. They offer several benefits, including easy isolation, low cost, widely available sources, and, most of all, their safety, since they are derived from sources consumed daily worldwide as foods. Researchers have reported PDEVs to prevent or treat diseases, including cancer, inflammation, and degenerative disorders [7,8,9,10,11,12]. This suggests that PDEVs could be one of the most promising medications to take into serious account in biopharmaceutical research. Specifically, the potential of PDEVs as natural or manufactured drug carriers is appealing, particularly for delivering medicines with specific therapeutic effects directly into damaged cells for disease treatment. A key advantage is the capacity of PDEVs to transport a range of therapeutic agents, including chemotherapeutic bioactives, DNA expression vectors, siRNA, and novel therapeutic molecules like antibodies, both in vitro and in vivo [13,14]. Significantly, PDEVs can be engineered for precise cellular targeting, indicating that these nanovesicles are exceptional prospects for the delivery of a diverse array of therapeutic medicines. It should be noted that PDEVs facilitate enhanced bioavailability of the secondary metabolites, which are generally known to have low bioavailability due to different issues such as low water solubility, reduced stability, and low ability to overcome natural body barriers [15,16,17,18]. Hence, it is a prerequisite that plant sources must be cultivated organically, free from synthetic agrochemicals, pesticides, and herbicide treatment, to guarantee that their PDEVs are devoid of chemical substances [19]. This is a significant benefit in utilizing PDEVs for many therapeutic applications, including nutrition, dermo-cosmetics, and therapeutics [20,21]. Furthermore, it has been demonstrated that PDEVs derived from identical fruits exhibit both qualitative and quantitative variations depending on whether they are sourced from intensive or organic agriculture [22]. Another significant advantage of PDEVs is their direct extraction from fresh fruits and vegetables, eliminating the necessity for large-scale cell factories required for human cell-derived exosomes, synthetic bioparticles like liposomes, or nanomaterials like metal and organic nanoparticles. This benefit can significantly lower production expenses [20,23].

It is now well-established through preclinical and emerging clinical evidence that PDEVs demonstrate therapeutic potential in preventing or treating diseases such as cancer, inflammation, and degenerative disorders. PDEVs’ utilizations are primarily focused on disease prevention and progression, using innate anti-inflammatory, antioxidant, and immunomodulatory cargos to hinder early pathogenic cascades. This preventive framework designates PDEVs as potential candidates for prophylactic measures both against and also in patients with pathological conditions [24,25]. This approach is in perfect harmony with the fundamental principle of Ayurveda, *Swasthasya Swasthya Rakshanam* (protection of health in the healthy), which emphasizes the preservation of *doshic* balance through seasonal routines (*Ritucharya*), individualized diet and lifestyle (*Dinacharya*), and phytotherapeutic interventions to prevent the onset of disease [26]. Ayurveda’s system for preventing disease is based on three main ideas: daily routines to keep *Vata*, *Pitta*, and *Kapha* in check; seasonal changes to adapt to changes in the environment; and herbal remedies to boost immunity (*Ojas*) and energy (*Bala*), which stop imbalances from turning into disease. In this management, plants play a pivotal role in modulating medicinal therapy. Ayurveda utilizes an impressive array of plants—more than 1200 species recorded in the classical literature, such as *Charaka Samhita* and *Sushruta Samhita*—in its formulations, harnessing their synergistic bioactives to restore balance when prevention is inadequate [26,27,28]. Thus, PDEVs could be an excellent candidate to bridge the gap between traditional preventive principles and contemporary nanomedicine, representing a state-of-the-art application of Ayurveda’s age-old phytotherapeutic expertise. Through the utilization of PDEVs’ inherent strength, modern research can not only reinterpret and support centuries-old methods but also open avenues for novel, bioavailable solutions that prioritize maintaining health first in a variety of applications comprehended in nutrition, preventive medicine, and therapeutics, and even cosmetics.

The current study concentrates on investigating the Ayurvedic plant sources utilized in PDEVs’ research, which are recognized for their substantial therapeutic activities. It also evaluates the formulation employed in Ayurvedic medicine for its limitations, which can be effectively addressed through the isolation of PDEVs. The current study, unlike other investigations, proposes Ayurvedic medicinal plants as an efficient and potential source of PDEVs. In conclusion, we advocate for PDEVs as a synergistic link between traditional Ayurvedic phytotherapy and modern nanomedicine.

## 2. Isolation and Characterization Techniques of Plant-Derived Extracellular Vesicles (PDEVs)

Plant-derived extracellular vesicles are fundamentally distinct from crude plant extracts in terms of structure, composition, extraction techniques, and functional characteristics. In contrast to heterogeneous plant extracts—derived from basic grinding, solvent extraction, or juicing—which produce polydisperse mixes of soluble phytochemicals (such as polyphenols and flavonoids), cellular debris, enzymes, and polysaccharides lacking vesicular order [29], PDEVs are intact, nanoscale lipid bilayer vesicles (often 30–150 nm) actively released by plant cells by multivesicular bodies and/or exocyst pathways. This membrane structure safeguards and enhances cargos, including miRNAs, proteins, lipids, and metabolites, facilitating tailored intercellular signaling and improved bioavailability [30]. This bilayer membrane stands as a characteristic feature when compared to other synthetic nanoparticles, organic nanoparticles, as well as plant extracts. The advantage of the isolation of PDEVs focuses purely on “concentrating the bioactive cargos into physiological spheric membranes” naturally produced by plant cells for intercellular communication without having bioactive preparations obtained by means of chemical moiety intervention [31].

### 2.1. Isolation of PDEVs

The isolation and purification of PDEVs can be accomplished using five standard methods. They are classified into five primary categories based on their separation principles: ultracentrifugation, ultrafiltration, immunoaffinity capture, size exclusion chromatography (SEC), and polymer precipitation techniques [32,33].

Differential ultracentrifugation is the most popular and oldest centrifugation method, since it is simple to use and gives a lot of results. The separation mechanism primarily relies on the principle that particles of varying sizes exhibit distinct sedimentation coefficients when subjected to centrifugal force. The first step is to use low-speed centrifugation (<10,000 g) to get rid of plant fibers and large cellular debris in the sample solution. As the speed and length of the centrifugation slowly rise, tiny vesicles are forced to the bottom by the high centrifugal force (>100,000 g) [15]. To obtain a pure collection of extracellular vesicles, density gradient separation is employed. After centrifugation, a density gradient made up of different sucrose solutions (8%, 15%, 30%, 45%, and 60%) is commonly made. Vesicles are sorted by size and density and mostly collected in the 30%/45% layer of sucrose solution [33], although it is time-consuming and not scalable. The ultrafiltration process can enhance the purification of PDEVs according to particle size or molecular weight. Small molecular particles will traverse the filter, whilst high molecular weight particles will be trapped by the membrane. Immunoaffinity isolation can further separate vesicles by employing specialized antibodies to capture appropriate protein markers on the EV surface, including tetraspanin proteins, CD63, CD9, and CD81, therefore preventing contamination from cytoplasmic proteins or RNA. The lack of specific antibodies that can recognize PDEVs’ surface proteins limits the use of immuno-affinity isolation. Size exclusion chromatography (SEC) is a technique for the separation of molecules or particles according to their size disparities. As the extract passes a chromatography column containing a porous resin stationary phase, vesicles will have distinct retention times due to their differing sizes. Consequently, when subjected to the elution buffer, PBS, soluble contaminants, including free proteins and tiny molecular compounds, are momentarily retained inside the column matrix, while larger extracellular vesicles rapidly traverse and are eluted in the initial fraction. Coprecipitation typically uses polymers, specifically Polyethylene Glycol (PEG), as a precipitation agent [32]. The hydrophilic PEG interacts with the water molecules surrounding vesicles, creating a hydrophobic microenvironment where EVs precipitate due to reduced solubility [34]. Table 1 summaries the techniques mentioned above in the PDEVs’ research.

A single current method for isolating and purifying PDEVs is significantly limited. Considering the properties of each separation technique, the majority of researchers employ a combination of the following procedures. Consequently, standardized isolation methods are essential for further investigations into the structure, functions, and biological properties of the PDEVs.

### 2.2. Characterization of PDEVs

A broad array of bioactive substances, including proteins, lipids, nucleic acids, and small-molecule metabolites, is encapsulated in their membrane structures and has a wide range of applications. The characterizations of PDEVs are essential for clarifying their roles and enhancing their clinical application. A standardized study of PDEVs always includes morphological characterization, physicochemical property characterization, and characterization of functional bioactive components.

Size distribution, zeta potential, and polydispersity index (PDI) are the primary indicators for assessing the stability of preparation procedures and batch-to-batch consistency of PDEVs. The size distribution and polydispersity index of PDEVs can be characterized using dynamic light scattering (DLS) analysis. Along with that, nanoparticle tracking analysis (NTA) provides information about the size and the concentration of individual extracellular vesicles in addition to precisely tracing their movements. It is hence beneficial for complex systems. NTA has been established as a benchmark method and a prerequisite for the characterization of extracellular vesicles. NTA analysis is critical when considering the scalability and reproducibility of PDEVs. Unlike DKS, NTA reports the particle size of the vesicle population rather than the hydrodynamic size of the vesicles. This allows for more accurate characterization of the extracellular vesicle. Nanoflow cytometry (NanoFCM) enables the sensitive investigation of several characteristics on a single particle; nevertheless, it requires fluorescent labeling and is costly to set up and run. To discover molecular markers, Western blotting can identify certain marker proteins in extracellular vesicles. Nevertheless, it is labor-intensive and time-consuming, and it is limited to recognized markers [35]. Zeta potential indicates the colloidal stability of PDEVs and allows for the assessment of their cellular membrane absorption by evaluating the polarity of the potential. Morphological characterization serves as the basis for identifying PDEVs, largely through the examination of vesicle morphology, size distribution, and integrity [36].

Presently, the primary methodologies for morphological characterization encompass transmission electron microscopy (TEM), scanning electron microscopy (SEM), atomic force microscopy (AFM), and cryogenic transmission electron microscopy (cryo-TEM). TEM is regarded as the benchmark for the morphological examination of PDVs, requiring negative staining techniques to distinctly elucidate the spherical or quasi-spherical structure, bilayer membrane attributes, and internal hollow morphology of vesicles. SEM is primarily used to evaluate the surface appearance and aggregation state of PDEVs, and it is frequently used in conjunction with TEM to thoroughly examine the structural properties of vesicles. Functional bioactive components are identified utilizing mass spectrometry and electrophoresis methods as the molecular foundation of PDEVs’ biological activity. For protein profiling, SDS-PAGE in conjunction with LC-MS/MS allows for thorough identification of protein composition; RNAseq and qRT-PCR enable precise quantification of RNA components in PDEVs; and GC-MS/LC-MS/MS enable the systematic characterization of small-molecule metabolites (such as terpenes, flavonoids, and phenolic acids) and lipid species (such as phospholipids, triglycerides, and sterols), which are essential for understanding functional mechanisms [37,38].

Standardized techniques for isolating and purifying PDEVs are crucial for maintaining consistency and efficacy in medicinal formulations. The current isolation techniques are time-consuming and have limited yields. It is necessary to have detailed investigations to fulfill the isolation of PDEVs on a large scale, while maintaining the quality of the vesicles. The lack of a consistent technique leads to significant variability in particle size, morphology, purity, and composition, even with the same plant source. Inconsistent isolation approaches cause heterogeneity, which is a significant challenge [39]. Furthermore, it is unclear if PDEVs isolated from distinct parts of the same plant have similar composition and therapeutic properties. Controlled genetic background, abiotic (light, temperature, and humidity), biotic (pathogens and probiotics), and processing factors should be considered to standardize the composition of PDEVs. Furthermore, the quality and origin of raw materials must be clearly defined [39]. The lack of biomarkers impacts the characterization and confirmation of isolated PDEVs, preventing the formation of a consistent nomenclature scheme [40]. Through established procedures for preparation, separation, and characterization, the quality control and storage for PDEVs guarantee purity, integrity, and functionality. In accordance with regulations and plant-specific consensus, these actions reduce pollutants, structural damage, and variability. Understanding the biological mechanisms of PDEVs is challenging because of their complicated makeup [41].

Hence, it is a prerequisite to study the quality control of the PDEVs with respect to their origin, sources, and biochemical and physical characterizations to maintain uniformity in the isolation and utilization of PDEVs for functional applications.

## 3. Background of Ayurvedic Herbal Preparations

Until the 19th century, Ayurvedic medications were likely made by practitioners in their residences. This approach persists in highly specific preparations transmitted through familial traditions. Domestic medicine preparation utilizing traditional techniques and methods is insufficient to satisfy the increasing demand for herbal products, both in quantity and in dose convenience. The 20th century, however, initiated efforts to industrialize Ayurveda. Currently, there are about 9000 traditional medicine production plants in the nation [42,43]. The employed procedures have primarily involved a direct mechanization of conventional processing apparatus or have been modified from the food, chemical, or pharmaceutical industries. This occasionally renders Ayurvedic production difficult and exhausting. The primary goal of traditional medicine research is to comprehend how medicinal plants can be employed to create novel herbal and allopathic medications. Understanding the fundamental processing techniques used in Ayurvedic medicine preparations opens a world of possibilities for improvements in pharmaceutical dosage forms [28,44,45].

The preparation can be categorized into primary and secondary dosage forms. The principal dose forms are *svarasa* (juice), *kalka* (bolus/paste), *kvātha* (decoction), *hima* (cold infusion), *phāṇṭa* (hot infusion), and *cūrna* (powder). They are the dosage forms in which the herbs are utilized directly. All these medications, except for cūrṇa, have a brief shelf life of up to one day. Secondary dosage forms include *vaṭi* (pills), *avaleha* (linctures), *sneha* (medicated oils/ghee), and *sandhāna* (fermented medications). These medicines possess an extended shelf life of up to one year. The preparation process follows two principal methods: (1) Extraction: membrane rupture and solute diffusion, including hot solvent, microwave-assisted solvent extraction, Soxhlet extraction, and ultrasound-assisted solvent extraction, and (2) Separation: volatility, adsorption, and size-exclusion for separation [45,46].

The manufacturing encounters considerable deficiencies attributed to inconsistencies in raw materials, the absence of standardization, and the selective use of chemical solvents. Along with these, the herbal constituent varies according to region, climate, harvest timing, and cultivation techniques, resulting in variations in active chemicals from batch to batch. Furthermore, pharmacologically significant medicinal plants demonstrate constraints such as poor single-drug delivery, low water solubility, low bioavailability, and poor targeting, resulting in limited clinical use. Adulteration or substitution with substandard botanicals is prevalent because of resource scarcity and inadequate verification [47]. Heavy metals (lead, mercury, and arsenic), microorganisms, pesticides, and fungal proliferation result from inadequate sourcing, processing, or storage. These concerns frequently result in variable potency, contamination hazards, and difficulties in clinical validation [48,49]. The main obstacles to the clinical use of Ayurvedic herbal medicinal plants include safety profiles, batch variability, production scalability, and regulatory frameworks. Shortages of medicinal plants brought on by overharvesting, habitat loss, climate variability, and inadequate cultivation infrastructure limit large-scale production. This results in erratic supply chains, rising raw material costs, production halts, and increased adulteration risks with inferior substitutes. Chromatographic fingerprinting and multivariate analyses show that batch-to-batch variability results in inconsistent active constituent levels due to geographical/climatic influences on phytochemical profiles, non-standard harvesting/processing protocols, and raw material heterogeneity [50]. Due to high cost and time scale, as well as inclusion/exclusion issues in comprehensive patient-centric approaches, the provisions in some markets circumvent stringent testing, making global harmonization and clinical trial design more difficult [51,52].

PDEVs can provide a modern alternative that can deliver bioactive cargos (as miRNAs, lipids, etc.) with enhanced stability, targeted uptake, and reproducibility from Ayurvedic medicinal plants [53]. PDEVs have been established in the field and exhibit significant advantages as follows: (i) their bilipid membranes preserve bioactives of cargo specific to their plant sources from external factors, such as pH fluctuations, thermal changes, and light exposure; (ii) naturally occurring nanovesicles that serve as effective tools for intercellular communication; (iii) they are immunologically tolerated, as they are present in foods commonly consumed by humans; (iv) they utilize a natural cellular uptake mechanism via membrane fusion, enabling them to traverse barriers, including the blood–brain barrier and the placenta; (v) they exhibit confirmed scalability, making them suitable for industrial applications; and (vi) they are non-toxic, originating from plants cultivated through organic farming practices and chemical-free isolation, which is entirely focused on physical (differential centrifugation and ultracentrifugation) method isolation [54,55,56,57,58].

## 4. Ayurvedic Plants as Sources of Plant-Derived Extracellular Vesicles

### 4.1. Plants Utilized for PDEVs Isolation

**Figure 2:**
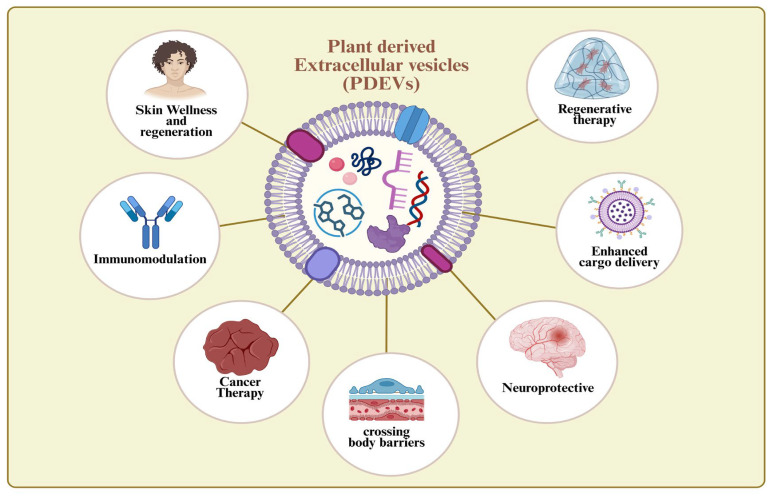
Therapeutic applications of PDEVs.

Plants used as curative medicines may represent valuable sources of PDEVs with great potential in health management. Figure 2 represents various branches of therapeutics touched by PDEVs. In fact, these plants are particularly enriched in specialized metabolomes, high levels of secondary metabolites, and diverse regulatory biomolecules, which are known to be selectively sorted into vesicular compartments. Experimental evidence has shown consistent vesicle size distributions, lipid bilayer architecture, conserved marker proteins, and functional cargo stability, supporting cross-species similarity in vesicle characteristics [59,60,61,62,63,64,65]. Given that bioactive compound biosynthesis and vesicle-mediated secretion are both linked to active metabolic and defense-related pathways, plants with established pharmacological profiles have a higher probability of yielding PDEVs with biologically relevant cargo [53]. Table 2 lists the plant sources associated with different health concerns.

**Table 2:** Overview of PDEVs’ botanical sources and their therapeutic potential for targeted diseases.

	Disease	Plant Sources	Common Name	Methods of Isolation	Effects	Ref.
**Digestive Health**	Ulcerative Colitis (UC)	*Portulaca oleracea*	Purslane, Kulfa, Loni	Differential Ultracentrifugation	Modulate the inflammatory microenvironment	[66]
		*Morus alba*	Mulberry	Differential ultracentrifugation followed by sucrose density gradient purification	Inhibits inflammation	[67]
		*Pueraria lobata roots*	Kudzu	Differential ultracentrifugation	Promotes intestinal tissue repair and modulate the immune microenvironment	[62]
		*Curcuma longa*	Turmeric, Haridra	Differential ultracentrifugation followed by sucrose density gradient purification	- Inflammatory suppressor, reduce oxidative stress in tissues - Regulate macrophage balance, protects intestinal barrier function, regulate the abundance of intestinal flora	[68,69]
		*Allium sativum*	Garlic, Lashuna	Differential Ultracentrifugation	Reduce intestinal inflammatory damage and regulate the abundance of intestinal flora	[63,70]
		*Centella asiatica*	Indian pennywort, Mandukaparni,	Enzymatic pre-processing + double ultracentrifigation + density gradient ultracentrifugation	Attenuate inflammatory responses and enhance the functions of immune cells in the intestinal Milieu	[71]
	Hepatocellular cancer	*Asparagus* spp.			Inducing apoptosis of cancer cells	[60]
		*Morus nigra* L. leaves	Black Mulberry, Krishna Toot	Sequential centrifugation		[72]
	Acute hepatic failure	*Allium sativum*	Garlic, Lashuna		Prevents liver inflammation decrease macrophage infiltration	[63]
**Musculoskeletal disorders**	Osteoporosis	*Pueraria lobata*	Kudzu	Differential Ultracentrifugation	Reduce osteoclastogenic factor, Block osteoblast calcification, improve osteoclast autophagy, Enhance bone differentiation	[73]
		*Morinda officinalis*	Morinda Root, Noni	Enzyme-assisted extraction followed by differential ultracentrifugation	Promotes MC3T3-E1 cell proliferation	[74]
		*Dioscorea* spp.	Yam, Suran	Differential Ultracentrifugation	Promote MC3T3-E1 cell differentiation and proliferation	[75]
			Ginseng	Centrifugation followed by sucrose gradient ultracentrifugation	Inhibition of osteoclast differentiation	[76]
	Osteogenesis	*Cissus quadrangularis*	veldt grape, Hadjod	Differential ultracentrifugation from plant callus	Ameliorate wounds and oxidative stress in the cells	[77]
	Muscle Atrophy	*Lycium barbarum*	Goji berries	Sucrose density gradient differential ultracentrifugation	Increase muscle grip strength	[78]
	Osteoarthritis	*Allium sativum*	Garlic	Differential Ultracentrifugation	Decreases inflammation	[79]
**Respiratory** **Diseases**	Acute Lung Injury	*Artemisia* spp.	Mug wort, Dhavanam	Sequential Ultracentrifugation	Decreases inflammatory cell infiltration while maintaining pulmonary immune equilibrium	[80]
	Pneumonia	*Houttuynia cordata*	Chameleon plant, Matsyagandha	Differential Ultracentrifugation	Inhibited Influenza A virus replication, reduce inflammatory factors, reduce lung inflammation	[81]
		*Pueraria lobata*	Kudzu	Differential ultracentrifugation	Regulate lung M1/M2 type macrophage balance	[62]
		*Zingiber officinale*	Ginger	Differential ultracentrifugation	Inhibition of cytopathic effects and reduce inflammatory factors	[82]
**Endocrine** **system**	Myocardial injury	*Momordica charantia* L.	Bitter gourd	Differential Ultracentrifugation	Ameliorates DOX-associated cardiomyocyte apoptosis and redox imbalance	[83]
	Type 2 Diabetes	*Vigna radiata* sprouts	Mung bean	Differential ultracentrifugation	Promotes glucose absorption and glycogen synthesis, reduce oxidative stress	[84]
		*Allium sativum*	Garlic	Differential ultracentrifugation followed by sucrose gradient purification	Stimulate outer membrane vesicle (OMV) release from *A. mucinophilus*	[65]
	Parkinson’s Disease	*Pueraria lobata*	Kudzu	Differential centrifugation combined with membrane filtration	Restores mitochondrial dysfunction and mitochondrial autophagy	[85]
		*Salvia officinalis* roots	Common sage	Differential ultracentrifugation	Preserved metabolic homeostasis, mitigated cellular oxidative stress and inhibited autoxidation	[86]
	Neuroglioma		Ginseng	Sucrose gradient ultracentrifigation combined with differential centrifugation	Promotes glioma cell apoptosis Inhibition of M2 macrophages	[87]
		*Momordica charantia* L.	Bitter gourd	Differential ultracentrifugation followed by sucrose density gradient purification	Penetrated the BBB and suppressed the glioma growth and metastasis	[88]
	Neuroblastoma	*Bacopa monnieri* L.	Waterhyssop, Brahmi	Differential ultracentrifugation	Suppress neuroblastoma cell growth and induce morphological alterations	[89]
	Breast Cancer	*Camellia sinensis* flowers	Fresh Tea	Differential ultracentrifugation	Stimulate ROS amplification and modulate gut microbiota.	[90]
		*Acorus calamus*	Sweet flag, Bach		Increases apoptotic activation	[91]
	Pancreatic Cancer	*Ocimum basilicum*	Basil, Sabja	Ultracentrifugation	Induces apoptotic ability	[92]
	Tumor regression	*Artemisia annua*	Sweet wormwood, Daman	Differential ultracentrifugation	Inhibit tumor growth and remolding the tumor microenvironment	[93]
		*Moringa oleifera*	Drumstick tree	Low-force filtration + centrifugatio	Exhibit proapoptotic effects of Hela and Jurkat cells	[94]
	Glioblastoma	*Citrus limon* L.	Lemon	Sequential centrifugation	Cytotoxic effects on tumor cells, minimizing endothelial toxicity and oxidative stress.	[95]
	Immunostimulatory	*Catharanthus roseus*	Periwinkle, Sadabahar	Differential ultracentrifugation	Promotes polarization and phagocytosis of macrophages as well as lymphocyte	[96]
		*Crocus sativus* flowers	Saffron, Kesar	Ultracentrifugation with density gradient	Selectively activate macrophages, increasing the expression of surface markers and pro-inflammatory cytokines	[97]
		*Solanum nigrum*	Black lampshade, Kakamachi	PEG-based precipitation	Decreased the expression of pro-inflammatory cytokine gene IL-6, and IL-6 protein	[98]
**Skin Diseases**	Melanoma		Ginseng	Differential ultracentrifugation	Induced macrophage polarization, improves tumoricidal function	[99]
		*Aloe* spp.		Differential ultracentrifugation	Activity by increasing oxidative stress in melanoma	[100]
	Skin Damage	*Dendrobium* spp.	Stalk orchids	Differential Ultracentrifugation	Improve inflammatory microenvironment	[101]
		*Triticum aestivum* L.	Wheat	Exo-spin™ exosome purification kit + size-exclusion chromatography (SEC)	Promote wound healing	[102]
		*Centella asiatica*	Indian pennywort, Mandukaparni	Differential ultracentrifugation	Improved skin hydration, elasticity and brightening	[61]
	Alopecia	*Allium sativum*	Garlic	Exo-spin™ Exosome Purification Kit	Stimulates hair follicle cell genesis and regeneration	[64]
	Wound Healing	*Nelumbo nucifera* leaves	Lotus	Compared all the isolation techniques, with tangential flow filtration (TFF) as optimal	Potential for wound healing, promotes the migration of HaCaTcells	[103]
		*Morinda officinalis*	Morinda roots	Enzyme-based cell wall digestion vs. Differential ultracentrifugation	Activates MAPK/YAP1 signaling pathway and accelerating wound healing	[104]
	Hair Growth	*Withania somnifera*	Ashwagandha	Differential ultracentrifugation	Increased secretion of VEGF-A from dermal papilla fibroblasts, increased anagen and decreased telogen hair rate	[105]

Aside from the plants currently studied, several Ayurvedic species remain unknown in the context of extracellular vesicle biology and constitute promising possibilities for future research.

### 4.2. Potential Ayurvedic Medicinal Plants for PDEVs Exploration

Edible fruits, medicinal herbs, and bioactive crop species of ayurvedic origins are among the various plant groups that may be considered to obtain PDEVs with similar structural traits but different molecular cargo. These source-dependent functional variations and cross-species vesicle structural similarities emphasize how crucial it is to methodically investigate several plant sources in order to find the best candidates for PDEVs’ purification. We analyzed some plants and the historical principles underlying their activity, which have the potential to be valuable sources for PDEVs and their potential applications in nutraceuticals, cosmetics, and therapeutics.

Ashwagandha (*Withania somnifera*), family Solanaceae, is popularly referred to as “Indian Winter Cherry” or “Indian Ginseng.” It is one of the most significant herbs in Ayurveda, the traditional medicine of India. The findings of some studies provide credence to the Ayurvedic theory of tonics, vitalizers, and rejuvenators, which suggests the clinical application of Withania somnifera in the prevention and treatment of numerous stress-related illnesses, including cancer, diabetes, arthritis, arteriosclerosis, premature aging, and hypertension [106,107]. Neural atrophy has also been identified as a major contributing factor in the etiology of patients with other neurodegenerative disorders, including Parkinson’s disease, Huntington’s disease, and Creutzfeldt–Jakob disease. Numerous studies demonstrate that Ashwagandha reduces, prevents, reverses, or eliminates synapse loss and neural atrophy. Consequently, Ashwagandha may be exploited to cure the above diseases and other neurodegenerative disorders at any stage, including prior to diagnosis, during the phase of moderate amnesia [108,109]. The advantageous impact of Ashwagandha root extract may be attributed to its GABA mimic properties. Ashwagandha and its ingredients, together with their metabolites, facilitate nerve development following a 7-day administration. Research substantiates the application of Ashwagandha as a mood stabilizer in clinical contexts of anxiety and depression [110,111]. Furthermore, Withaferin A and 3-b-hydroxy-2,3-dihydrowithanolide F, extracted from Ashwagandha, have antibacterial, antitumoral, immunomodulatory, and anti-inflammatory effects, which serve in the prevention of numerous diseases [112].

Shatavari (*Asparagus racemosus*), family Asparagaceae, is a prominent medicinal plant recognized for its adaptogenic, immunomodulatory, anti-inflammatory, antioxidant, antibacterial, and gastroprotective properties. It is abundant in bioactive chemicals, including steroidal saponins (shatavarins), flavonoids, alkaloids, and polysaccharides, which enhance its therapeutic efficacy [113]. It has been extensively utilized in Ayurveda for female reproductive health, lactation assistance, digestive wellness, and stress alleviation [114,115,116]. Research substantiates its efficacy in addressing hormonal imbalances, stomach ulcers, diabetes, and neurological diseases. In addition, it possesses diuretic, antidiarrheal, and analgesic properties. By reducing oxidative stress, controlling neuroinflammation, and inhibiting the formation of amyloid beta, it has been shown to exhibit neuroprotective effects that can lessen the pathogenesis of Alzheimer’s disease [117,118].

Giloy (*Tinospora cordifolia*) is a herbaceous vine of the family Menispermaceae. The medicinal properties of the Tinospora plant are attributed to its phytochemical elements, including alkaloids, flavonoids, glycosides, aliphatic compounds, diterpenoids, vitamins, tannins, lactones, steroids, coumarins, lignans, triterpenes, and nucleosides [119,120]. The alkaloid-rich fraction from the stem, comprising palmatine, jatrorrhizine, and magnoflorine, has been documented to exhibit insulin-mimicking and insulin-releasing effects both in vitro and in vivo. Isolated alkaloids from the plant exhibited an insulin-related response in the production of hypoglycemic action [121]. The phytoconstituents extracted from Giloy, including magnoflorine, tinocordioside, 11-hydroxymuskatone, cordifolioside A, N-methyl-2-pyrrolidone, and N-formylannonain, exhibited cytotoxic and immunomodulatory properties. Isolated phytoconstituents augmented the phagocytic capacity of macrophages, enhancing nitric oxide (NO) synthesis through splenocyte activation and increasing the generation of reactive oxygen species (ROS) in neutrophil immune cells [122]. The anticancer efficacy of T. cordifolia has been documented against multiple tumors or malignancies. Ethanolic extracts from all sections of the plants demonstrated hepatoprotective effects against carbon tetrachloride-induced liver injury in rats. The extracts demonstrated an osteoprotective activity in vitro in human osteoblast-like MG-63 cells and primary osteoblasts obtained from rat femurs [123]. Additionally, it is conventionally employed in the management of asthma, and its juice is utilized for addressing persistent coughs [121]. The aqueous extract of T.cordifolia mitigates mast cell-mediated allergy responses in rats through antihistaminic properties. Furthermore, reduced symptoms of allergic rhinitis, including sneezing, nasal discharge, nasal obstruction, and nasal pruritus, were also documented [124].

Manjishtha (*Rubia cordifolia*) is a climber herb with little greenish-white blooms gathered around purplish, meaty fruits. It is known for its hepatoprotective, anti-oxidative, cardioprotective, gastroprotective, anticancer, nephroprotective, and wound healing activities [125]. Mayurshikha (*Actiniopteries dichotoma*) is a pretty fern, possesing anti-inflammatory, antihistaminic, antimicrobial, antihelmintic, anticholinergic, analgesic, and antitubercular activities [126]. Yashtimadhu, Liquorice (*Glycyrrhiza glabra*), commonly known as liquorice, which is utilized to address respiratory conditions (cough and asthma), improves digestion by alleviating ulcers and acidity, and strengthens immunity. It serves as an anti-stress agent, enhances skin health by diminishing pigmentation, and improves cognitive performance [127]. Owing to these advantages, Chavan et al. studied the lipid-based ayurvedic formulation of liquorice to analysis chromatographic and stability study with the intention to enhance the shelf life of the formulation [128]. Dhatki Kurz (*Woodfordia fruticosa*) is classified within the Lythraceae family. This plant contains several isolated phytochemicals, including tannins, flavonoids, anthraquinones, glycosides, and polyphenols. The extracts of its flowers and leaves are linked to beneficial medicinal effects. These phytochemical substances possess numerous pharmacological activities, including antibacterial, hepatoprotective, cardioprotective, antioxidant, antiulcer, analgesic, antihyperglycemic, immunomodulatory, and anti-tumor effects [129]. Tulsi (*Ocimum* *sanctum* L. and *Ocimum tenuiflorum* L.) from family Lamiaceae is usually referred to as holy basil. It possesses anti-inflammatory, antioxidant, antidiabetic, and anti-stress properties, attributed to bioactive substances such as eugenol, rosmarinic acid, and ursolic acid. Tulsi demonstrates adaptogenic properties via regulating the HPA axis, decreasing cortisol levels, and improving stress resilience in rodent models, akin to antidepressants. Leaf extracts exhibit antibacterial activity, anticancer potential through the activation of apoptosis and suppression of angiogenesis, and antidiabetic action by the inhibition of α-glucosidase. The anti-inflammatory effects compete via NF-κB inhibition, while hepatoprotective and radioprotective functions arise from the overexpression of antioxidants (SOD and CAT). Tulsi targets lifestyle diseases, an adjuvant in type 2 diabetes mellitus (with glibenclamide), metabolic syndrome, psychological stress, and infections. Organic cultivation increases eugenol and terpenoid yields, facilitating nutraceutical synthesis [130,131,132]. Shankhapushpi Choisy (*Convolvulus pluricaulis*) is recognized for its neuroprotective, anti-inflammatory, and antioxidant properties for enhancing cognitive functions and supporting mental health. Secondarily, anticonvulsant, antithyroid, antihyperglycemic, hypolipidemic, and analgesic effects are reported for this plant [133]. Other numerous plant sources are Amla or Indian Gooseberry (*Phyllanthus emblica*), Haritaki or Chebulic Myrobalan (*Terminalia chebula*), Beheda or Beleric myrobalan (*Terminalia bellirica)*, Arjuna (*Terminalia arjuna*), Bhringraj, Eclipta (*Eclipta prostrata*), Hibiscus *(Hibiscus sabdariffa*), *Olibanum (Boswellia serrata*), Babool (*Acacia nilotica*), *Neem* (*Azadirachta indica*), which is known to be a blood purifier and has antimicrobial properties, Bael (*Aegle marmelos*), *Cyperus rotundus*, and many more [134].

In spite of the abovementioned positive effects of plants like Ashwagandha, shatavari, and others being exploited by ethnobiological research and industry, their use in PDEVs’ research is limited to a few reports. They result from ethanolic extraction, large-scale solvent manufacturing, and variable batch consistency [45]. In summary, the combined biological, biochemical, and pharmacological attributes of the chosen plant sources robustly endorse their potential as candidates for the extraction and examination of PDEVs [20]. Diverse plant sources would be inherently able to produce extracellular vesicle populations, as evidenced by the growing understanding of vesicle secretion in plants as a conserved and physiologically significant process connected to endosomal trafficking, membrane remodeling, and stress-responsive signaling. Since PDEVs are known to be selectively incorporated into vesicular cargo and may directly influence downstream functional consequences, plants that are rich in secondary metabolites, regulatory biomolecules, and bioactive lipids are especially appealing in this context [19]. PDEVs would be helpful to have an enhanced delivery of the secondary metabolites and the cargo from the sources when compared to the plant extract [22]. While species-specific variations in vesicle yield, size distribution, surface makeup, and molecular loading are expected, this diversity offers a strategic benefit for application-focused selection and source optimization. A systematic and comparative assessment of these plant possibilities would enhance the comprehension of PDEVs’ biogenesis and cargo heterogeneity, while also aiding in the identification of source-dependent functional markers [56]. Consideration of these plants as a source of PDEVs would be interesting and synergistic to its existing effects. This source-guided method should speed up the creation of PDEVs’ platforms that can be used for therapeutic delivery, immunomodulation, and regenerative research that are repeatable, biologically active, and suited to specific applications.

## 5. Future Perspectives

Ayurvedic medications, while proven to be effective for centuries, may have problems related to the way of their preparation and their standardization in terms of cargo and physicochemical features. A key point is that the currently used ayurvedic preparations are obtained through an alcoholic method of extraction. This reduces the level of bioavailability of the plants’ content. More research is needed to obtain effective and safe products. Unquestionably, Ayurvedic medicinal herbs have been used successfully to prevent and treat a wide range of ailments, and in preventive medicine, healthcare, and rehabilitation. However, the constraints of traditional preparations, such as poor single-drug delivery, low water solubility, lower bioavailability, and poor targeting, have greatly limited the clinical use of pharmacologically essential medicinal plants. The extraction of PDEVs from traditional Ayurvedic medicinal herbs aims to augment medical efficacy by enhancing activity, bioavailability, and targeting, hence improving the shortcomings of conventional formulations. A few considerations must be seen:

(1) The effectiveness of the extracted extracellular vesicles differs based on the specific plant sections, requiring the identification of the most efficient part. The isolation of PDEVs should be considered while aiming for targeted utilizations.
(2) Since traditional herbal medicines undergo specific processing, it remains unclear whether PDEVs extracted directly from source plants can fully replicate their therapeutic benefits. Addressing this gap requires comprehensive characterization, methodological standardization, and formal validation of these PDEVs.
(3) Given that PDEVs’ biogenesis entails the selective scavenging and encapsulation of bioactive metabolites, nucleic acids, and proteins from their sources into extracellular release, it is imperative to source plant materials from organic farming to avoid contamination by synthetic agrochemicals, pesticides, and herbicides commonly used in either conventional or intensive farming.
(4) It is still not clear whether using plant extracellular vesicles together can have the same effects as herbal medicine. It is also not clear whether using PDEVs from these sources will cause adverse immunogenic responses when compared to traditional preparations.

However, previous results on PDEVs’ preparation, obtained with either single or mixed fruits and vegetables, have shown that PDEVs have always demonstrated a superior efficacy when compared to commercially available bioactive molecules [135,136]. Moreover, we know that PDEV-based products are currently used in Italy as nutraceuticals and sold in pharmacies with no evidence of side effects. This, of course, is an important guarantee for the setup of new PDEV-based products.

With PDEVs, we should consider that their content in a single biomolecule may be increased by artificially uploading it. In fact, PDEVs are considered the ideal delivery system for any kind of molecule, due to the natural property of EVs to deliver any molecule through the body and between different species [137]. The current study proposes PDEVs as a synergistic link between traditional Ayurvedic phytotherapy and modern nanomedicine. This would surely supply solutions in translational research. Isolating PDEVs out of Ayurvedic medicinal plants will probably be intriguing in scaling up for the global healthcare sector.

## Figures and Tables

**Table 1:** The isolation techniques utilized in PDEVs’ research.

Technique	Principle	Advantages	Drawbacks
Ultracentrifugation	Size selective sedimentation	High particle yield; methodological standardization	Time-consuming, protein co-pelleting
Density gradient centrifugation	Buoyant density separation	Exceptional purity	Long runtime, equipment intensive and non-scalable
Ultrafiltration	Molecular weight cutoff filtration	Scalable and rapid	Membrane fouling, shear-induced vesiculation
Immunoaffinity	Antigen–antibody surface recognition	Marker-specific enrichment, high specificity	Costly reagents; low yield, epitope masking
Size Exclusion chromatography	Hydrodynamic radius-based elution	Preserved integrity, polydispersity reduction	Requires dilution, column capacity limited
Polymer precipitation	Hydrophobic/salting-out aggregation	Rapid, no complex instrumentation required	Polymer residues, compromised purity

## Data Availability

No new data were created or analyzed in this study.
